# Assessing the experience of using synthetic cannabinoids by means of interpretative phenomenological analysis

**DOI:** 10.1186/s12954-017-0138-1

**Published:** 2017-02-10

**Authors:** Szilvia Kassai, Judit Nóra Pintér, József Rácz, Brigitta Böröndi, Tamás Tóth-Karikó, Kitti Kerekes, V. Anna Gyarmathy

**Affiliations:** 1 Doctoral School of Psychology, Eötvös Loránd University, Budapest, Hungary; 2 Institute of Psychology, Eötvös Loránd University, Budapest, Hungary; 3 Institute of Psychology, University of Szeged, Szeged, Hungary; 4 Faculty of Health Sciences, Semmelweis University, Budapest, Hungary; 5 Johns Hopkins Bloomberg School of Public Health, Baltimore, MD USA

**Keywords:** New psychoactive substances, Synthetic cannabinoids, User experience, Harm reduction, Interpretative phenomenological analysis, Qualitative study

## Abstract

**Background:**

New psychoactive substances (NPS) have been increasingly consumed by people who use drugs in recent years, which pose a new challenge for treatment services. One of the largest groups of NPS is synthetic cannabinoids (SCs), which are intended as a replacement to cannabis. While there is an increasing body of research on the motivation and the effects associated with SC use, little is known about the subjective interpretation of SC use by the people who use drugs themselves. The aim of this study was to examine the experiences and personal interpretations of SC use of users who were heavily dependent on SC and are in treatment.

**Methods:**

A qualitative research method was applied in order to explore unknown and personal aspects of SC use. Semi-structured interviews were conducted with six participants who had problematic SC use and entered treatment. The research was conducted in Hungary in 2015. We analyzed data using interpretative phenomenological analysis (IPA).

**Results:**

Participants perceived SCs to be unpredictable: their initial positive experiences quickly turned negative. They also reported that SCs took over their lives both interpersonally and intrapersonally: the drug took their old friends away, and while initially it gave them new ones, in the end it not only made them asocial but the drug became their only friend, it hijacked their personalities and made them addicted.

**Conclusions:**

Participants experienced rapid development of effects and they had difficulties interpreting or integrating these experiences. The rapid alteration of effects and experiences may explain the severe psychopathological symptoms, which may be important information for harm reduction and treatment services. Since, these experiences are mostly unknown and unpredictable for people who use SCs, a forum where they could share their experiences could have a harm reducing role. For a harm reduction point of view of SCs, which are underrepresented in literature, it is important to emphasize the impossibility of knowing the quantity, purity, or even the number of different SC compounds in a particular SC product. Our study findings suggest that despite the adverse effects, including a rapid turn of experiences to negative, rapid development of addiction and withdrawal symptoms of SCs, participants continued using the drug because this drug was mostly available and cheap. Therefore, a harm reduction approach would be to make available and legal certain drugs that have less adverse effects and could cause less serious dependence and withdrawal symptoms, with controlled production and distribution (similarly to cannabis legalization in the Netherlands).

## Background

New psychoactive substances (NPS) have been increasingly used by people who use drugs in recent years, which poses a new challenge for treatment services and researchers [1]. NPS are sold as replacements to illicit drugs, but they often contain unknown compounds. They are produced in small laboratories or on a commercial scale in clandestine factories by organized crime groups [2]. One of the largest groups of NPS is synthetic cannabinoids (SCs), which are intended as a replacement to cannabis [3]. SCs appeared on the drug market in the mid-2000s and were sold as herbal smoking mixtures; since then, hundreds of different compounds have appeared [2].

In Hungary, NPSs appeared in 2010 and rapidly dominated the illicit drug market [4]. The number of seizures of SC—also known as “herbal”, “bio-weed”, or “sage”—was nearly double the number of seizures of herbal cannabis in 2014. The range of substances found in the products follows the changes in legislation: between one and two dominant active substances could be found on the market in each individual period. After the individual substances had become regulated, their presence on the drug market dropped considerably and their places were taken over by new substances (that were not yet regulated) within 1–3 months in the period of 2011–2014 [5]. The dynamics of these processes changed in 2015, as the scope of the substances that could be traded without any criminal consequences was narrowed drastically by the expansion of the generic regulation. By the end of the year, the place of ADB-FUBINACA, which was legal until then and dominant in seizures, was overtaken by AMB-FUBINACA and 5F-AMB, regardless that these substances had already been controlled since October 2014 (in Hungary substances are banned compound-by-compound [5]). Users obtain drugs from acquaintances and friends or from the internet [5].

Motivations to use SCs include their easy availability, legal status, low price, and inability to be detected by standard drug tests [6–8]. Other motivations to use include the wish to experience pleasant feelings, recreational effects, and relaxation [9, 10]. The lack of safety information may lead to the incorrect assumption that SCs are safe [11, 12]. Compared to cannabis use, the consumption of SCs may be associated with more adverse and unpredictable physical and psychological effects [10, 13–15], although people who use SCs reported subjective experiences that are similar to the use of cannabis, and they also described unique effects that are very different from other kind of drugs [12]. In addition, in the study of Winstock and Barratt [16], the effects of SCs last for a shorter time and they are more intense compared to cannabis, and many undesired effects have also been described by participants.

In a study by Vandrey et al. [12], 87% of people who use SCs reported having positive effects (e.g., they felt a pleasant high, stimulated, and energetic), but 40% reported negative or unwanted effects (e.g., dry mouth, heart racing, and paranoia). A subset of respondents felt unable to cut down or stop SC use (38%), experienced tolerance (36%), used for longer periods than originally intended (22%), and had interference with other activities (18%). Barratt et al. [9] found that 68% of the people who used SC reported at least one side effect, such as decreased motor coordination, fast and irregular heartbeat, dissociation, dizziness, and psychosis.

Winstock et al. [17] conducted a research study among people who used SC who sought emergency medical treatment following their SC use. They found the relative risk of severe side effects associated with use of SCs to be 30 times higher compared to that of cannabis (SC is a full agonist and THC is a partial agonist of the CB1 receptor [18, 19]). Respondents reported more adverse symptoms after the consumption of SCs versus cannabis, including panic, paranoia, anxiety, and aggression, and they used emergency services more often as well.

There is a growing body of literature on clinical case reports about severe consequences of SC use. The consumption of the drug leads to emergency room visits, though clinical treatment often remained short and symptomatic [13, 15]. Castaneto and colleagues [13] conducted a literature review on acute SC intoxication and found that patients reported that intoxication occurred within 2–5 h and lasted for about 24 h. Treatment to relieve symptoms included benzodiazepines and intravenous saline solution. In a systematic review, Tait et al. [20] summarized the adverse events arising from SC use. They found that major complications included cardiovascular events, acute kidney injury, generalized tonic-clonic seizures, psychiatric presentations, and hyperemesis, and typically involved young males with tachycardia, agitation, and nausea requiring only symptomatic care with a length of stay of less than 8 h. High intoxication level was reported by cannabis users who reported floating feelings, being drowsy, a sensation of time alteration, less sociability, more talkativeness, worsening memory, inability to think clearly, paranoia, increased sexual pleasure, sleep difficulties, hallucinations, and decreased sexual drive [21].

According to clinical case reports, the withdrawal symptoms of SCs are similar to cannabis but more severe [22–24]. Withdrawal symptoms including agitation, irritability, anxiety, and mood swings were reported by people who used SCs [25]. In a study by Van Hout and Hearne [26] that examined the experience of SC withdrawal, participants described intense cravings, compulsive all-consuming seeking, use and re-dose behaviors, and a fear of the psychiatric and self-harms caused during withdrawal. Cannabis dependence syndrome could occur with heavy chronic use in individuals who report problems in controlling their use and who continue to use the drug despite experiencing adverse personal consequences [27]. Wiesbeck et al. [28] conducted a study in a large population to evaluate marijuana withdrawal symptoms. Almost 16% of the most frequent marijuana users (who had used the drug daily for an average of almost 70 months) experienced withdrawal syndrome. These symptoms included nervous tense, restlessness, sleep disturbance, and appetite change.

Every-Palmer [29] examined psychosis among people who used SC and found that anxiety and psychosis symptoms were reported after SC use and lasted between 2 days and several weeks. Müller et al. [30] reported a case where a patient’s psychotic symptoms that had developed as a result of prior cannabis consumption not only worsened after subsequent SC use but the patient also started experiencing auditory and paranoid hallucinations that he never had before. Bassir et al. [31] compared clinical presentations of SC users with cannabis users in a psychiatric inpatient setting and found patients who had smoked SC where most likely to experience psychosis, agitation, and aggression than those who only smoked natural cannabis.

Bilgrei [32] analyzed discussions on experiences of SC use in posts of an online drug forum and in interviews with forum participants. The study illustrates the process of alteration of experiences from positive to negative during the consumption of SCs. While there is an increasing body of research on the motivation and the effects associated with SC use [6–10] and Bilgrei [32] examined experiences based on the forum participants’ accounts, little is known about the subjective interpretation of SC use by the people themselves who used SCs. The aim of this study was to examine personal interpretations of experiences derived from the use of SCs. Meshack et al. [7] suggests that qualitative research offers an excellent opportunity to uncover subjective aspects of personal motives and social norms in connection with drug consumption. Therefore, the aim of our study was to assess the experiences of SC use and analyze subjective interpretation of experiences of people who had problematic SC use, by means of interpretative phenomenological analysis (IPA), a qualitative research tool that works with a person-centered approach [33].

## Methods

### Participants

The current study was conducted in two Hungarian drug rehabilitation centers that work with a recovery approach and require abstinence. The participants attended the treatment voluntarily. Based on the methodology of IPA, a purposive sample was recruited [33] among the treatment participants. According to the methodology of IPA, the idiographic inquiry [33] requires a homogenous and small sample. According to Smith et al. [33], the recommended sample size for an IPA study is three to six interviewees. Therefore, the current study involved six male patients (aged 20–27 years) who were self-identified SC users. No female users were available. Before the analysis, they had been using SCs for at least 2–6 years, and at the time of the study they had been abstinent for at least 1 month. It was assumed that SCs were the dominant components of the substance that they smoked. The study focused on a particular sub-group of the SC user population: people who had problematic SC use and entered treatment. Due to their abstinence, the effects of the drug did not influence participants’ responses, and they could describe their experiences also from an outsider’s perspective. Additionally, by using IPA, the researcher could examine processes: how experiences could change over time [33] and over the addiction process, as many previous IPA studies explored experiences of people who used drugs in recovery [34–36].

The participation in this study was voluntary, and we use pseudonyms for the participants to protect their identity. Information about their sociodemographic characteristics are presented in Table 1. The Institutional Review Board at Eötvös Loránd University approved all study protocols.

**Table 1:** Participants’ sociodemographic characteristics. (Participants’ names have been changed to protect their identities)

Name	Age	Sex	Marital status	Highest educational attainment	Duration of SC usage (year)	Time spent in treatment
Ricsi	27	Male	Single	High school	2	1 year
Attila	20	Male	Single	Elementary school	3.5	1 month
Zsolt	23	Male	Single	High school	6	2 months
Levente	22	Male	Single	Elementary school	6	1 month
Jerob	20	Male	Single	Elementary school	2.5	3 months
Szilveszter	21	Male	Single	Elementary school	2	6 months

### Data collection

For this study we conducted semi-structured interviews by using open-ended questions. The interviews lasted 45–60 min. In IPA studies, participants are perceived as experts on the subject, and therefore the interview schedule should allow ample opportunity for them to tell their stories, and should be flexible enough to go into novel areas and produce richer data [37]. The interview schedule contained the following questions (which were modified in the light of participants’ responses; [37]): “Tell me about your experiences of SC use”, “How did you see yourself, when you used the drug?” How did others see you, when you used the drug?” “How are the experiences of SC use are different from using other drugs?”

### Data analysis

The interviews were transcribed verbatim, and we analyzed data using IPA. During the analysis, we applied the aspects of IPA: the accounts of six participants were detailed enough to track the participants’ sense-making of their experiences. IPA works with “double hermeneutics”, where the participants try to interpret their experiences, and the researcher tries to interpret the participants’ interpretation of their experiences [33]. During the analysis, initial notes or comments were added upon close and multiple readings of the interview transcripts. Through making initial notes and comments, the researcher captured the meaning of the experience in each participant’s accounts. During this deep and intense analytical work (double hermeneutic), which is often explained by the “hermeneutic cycle” (the researcher steps into the participants’ meaning making process and analyzes it from an interpreter’s perspective, see [33]), “emergent themes” are formed. In the second stage, patterns and themes across the “emergent themes” are identified and clustered into more abstract “master themes” [33, 37]. SK, JNP, and JR determined the themes and the emerging themes and reach consensus. Themes grouping to emergent themes were revised by the second group of authors (BB, TTK, KK, VAG). A consensus was then reached regarding the emergent themes. According to Rodham et al. [38], the reliability could be ensured during the IPA analysis by the shared analysis of researchers. The analysis focused on participants’ interpretations of their experiences derived from the use of SCs. The emergent and master themes are presented in Table 2.

**Table 2:** Emergent and master themes

Master themes	Emergent themes
1. SCs are unpredictable	Unpredictable effects
	Rapid alteration of experiences from positive to negative
2. SCs take over people’s lives	Interpersonal context: SCs both take away old friends and give new ones
	Interpersonal context: becoming asocial
	Interpersonal context: the drug becomes a friend
	Intrapersonal context: the drug hijacks the personality

## Results

Participants in this study interpreted their personal experiences of using SCs. Due to the novel effects of the drug, they perceived SCs to be unpredictable (first master theme) and that the drug took over their lives (second master theme).

### SCs are unpredictable

#### Unpredictable effects

Participants started using SCs by recommendations of others in their user group or this was the first drug they have tried. They reported that the effects of the drug were very different from those of other drugs; therefore, they perceived the effects of SCs to be unpredictable and that big differences in the effect could be observed at each drug consumption episode.

Everybody has different experiences about it, when I smoke, I feel normal, I feel it a little bit inside, but I do not look different. And he (a friend) smoked once and passed out for at least two hours (Ricsi)

According to their accounts, the impact of the drug on the user was also unpredictable. They did not experience this unpredictability about themselves in case of other drugs.

When I used mef (mephedrone) I got the same feeling as I had when I was young. I was in a good mood, I could talk to anyone, I was good with everyone, and I was just talking and talking…the bio (bio-weed) is dangerous I cannot realize myself, I don’t care about anything. This is illusory, it can change you, it can make you sick, anything can happen, you can go crazy, and you can do things that destroy your life. (Jerob)

SCs are described as being unpredictable due to the novel effects, which could be different in comparison to the previous SC experiences and experiences of using other drugs. Participants mentioned multiple unpredictable physical and psychological effects. Even after prolonged use, SCs could still evoke some unusual experiences, which are more intense and faster than in case of cannabis and other drugs.

Cannabis is usually… two and a half hours… the bio-weed has a forty-five–minute effect at most. This is a big difference. But it would be impossible to tolerate two and a half hours of this kind of effect that the bio-weed has. This experience is brutal. (Szilveszter)

Some participants’ perception of the drug was paranoid, and they used metaphors to express their uncertainty of unpredictable effects of the drug and their vulnerability against it. They often characterized it as a danger or compared it with a virus or an epidemic.

This is a kind of virus or epidemic, or I don’t know what… it can infect everyone and can take anyone to the bottom (Jerob).

#### Rapid alteration of experiences from positive to negative

SCs are described as being unpredictable, because at the beginning, they had some positive effects, of which participants mentioned relaxation and recreational aspects. But after a few consumptions, their experiences rapidly turned negative, and addiction appeared. Then the aim of consumption was no longer to reach the positive effects, but to avoid withdrawal symptoms. The participants continued the use of the drug in spite of negative experiences due to the rapid appearance of addiction and also because this drug was mostly available.

At the beginning you can eat more, you are in a good mood… you can see positive things. But later it turns into its opposite, I couldn’t eat, I was lazy, even if I had the drug. (Attila)

I had positive experiences about this, but as time went by, I saw many disadvantages of it. (Zsolt)

I felt I became addicted…I couldn’t sleep, I smoked it in vain, because I woke up in every hour in the night to smoke until morning. When I woke up in the morning my first thoughts were about how can I get some more again. (Levente)

### SCs take over people’s lives

The participants reported fast alteration of their experiences from positive to negative and felt that they had lost control over their behavior as well as their physical and psychological conditions. Due to these unpredictable effects, they felt the drug hijacked their lives. The hijacking effect of the drug was perceived in both interpersonal and intrapersonal contexts.

#### Interpersonal context: SCs both take away old friend and give new ones

Interviewees described that through using the drug they got involved with a company (a user group) that gave them a sense of belonging, though relationships had a purpose; they meant a sure source to obtain the drug.

I belonged to a group where I didn’t want to belong, but I was there, because the drug was there. (Zsolt)

Later participants realized that these relationships were worthless, but at the same time they lost their other relationships (such as family and friends), and they perceived the drug took them away, due to the turning inward and isolative effects of SCs.

Sometimes we didn’t think about it, that we can hurt people, who really loved us. People who raised us and always were there for us. (Levente)

#### Interpersonal context: becoming asocial

Social and personal effects of the drug that participants reported included turning inward and becoming asocial. As this happened against the participants’ will, they perceived that the drug hijacked them. In these accounts a process emerged, where first the drug gave new friends, but later it gradually took it away, because it strengthened participants’ egoism and disinterest in social connections. They retreated from their social world, hid in their room, and preferred to use SCs alone.

I started to use the drug with my friends, then I became completely asocial. So, I bought my bio-weed and I smoked it at home at night. (Zsolt)

When I smoked the bio-weed, I plunged in my earphone and the whole world switched off, and there was only me. During those times I did not like talking to anyone. (Ricsi)

When I smoked I was wallowing in self-pity, I felt sorry for myself, I was alone, I didn’t care about anybody else, I hated everyone. (Jerob)

#### Interpersonal context: the drug becomes a friend or a partner

Participants perceived the drug as a friend or a partner, which—even though it made them turn inward—could help overcome loneliness. “I was so lonely,… bio’ was my friend, because it was always there, when nobody else was, it always made it possible to be there for me.” (Jerob)

In later stages of participants’ drug use carrier, SCs become the most important thing and the only thing that they perceive. Participants withdraw from their social world and everyday life, and all their thoughts and activities focus on SC consumption.

You become unconcerned about your things, for example your clothing, your hair, and etc. you don’t care about these things, only to have the drug. When I woke up I smoked, if I had it. If I didn’t have the drug, I roamed until I got some (Attila)

On the other hand, SCs become an elementary need, a basic part of life, without which withdrawal symptoms appear: “When I had no money or possibility to get some, it felt like I was going to die without it. It was to me like food or water for normal people.” (Zsolt)

#### Intrapersonal context: the drug hijacks the personality

Participants mentioned the strong impact of SCs on their mental states, more specifically a temporally change in consciousness and behavior that lead to losing control. This is why they felt they were hijacked by the drug.

During that time (of consumption), I felt like my body was controlled by someone else, like it wasn’t me, I couldn’t control it. (Levente)

At the end I sank into it, I couldn’t remember what I did ten minutes before, if someone asked me about it, I couldn’t answer it, so it influenced my life so badly. (Attila)

The drug totally distorted my personality, it turned myself inside out… it made me blunt, and switched off my brain. (Zsolt)

By saying that the drug hijacked them, participants tried to describe their experience of addiction, which was perceived as compulsive drug use.

You become blunt, like if you don’t know about yourself, your body desires the drug so much, so you smoke. You know it is bad, but you want the drug and it wants you, it is not good, but you smoke it compulsively. (Attila)

It is impossible to distinguish the experiences of drug use and addiction in these accounts. Due to the rapid development of addiction that was reported by the participants, the experiences of drug use are the same as the experience of addiction; thus, participants mentioned predominantly negative experiences.

I think this bio-weed causes addiction the fastest, because I have not experienced this kind of addiction before, not with any other drug. (Levente)

Participants often mentioned their first thoughts in the morning were all about the drug: “When I opened my eyes, it was already prepared around me in the bed: the filter, the paper and fresh tobacco” (Ricsi). And every thought they had was about the acquisition and consumption of the drug: “In the end I was so addicted that I went to bed with it, I woke up with it every hour, and I was unconscious, and then I woke up to realizing that I was smoking it.” (Attila)

Description about both the addiction of the body and psyche emerged in the accounts, especially in presentations of withdrawal symptoms. The addiction of the psyche became apparent in anxiety attacks, craving, feeling of guilt, and excruciating desire for the drug, which the participants perceived as the drug hijacking their thoughts. The addiction of the body was defined by withdrawal symptoms including tremor, passing out, and insomnia. In both cases, participants felt unable to control the symptoms and their addiction, so they perceived being vulnerable.

I was sweating, I couldn’t sleep, my nap was numbing, I had many physical effects… I desired the drug more and more, I became stressful, I became neurotic, and at the end I could not live without it. (Attila)

I smoked at night and I fell asleep, two hours later my body woke me up to smoke again. (Zsolt)

## Discussion

In this study we assessed the experiences of SC use. During the analysis we utilized IPA, a qualitative research method that is able to assess personal experiences and examine how the participants interpret a particular experience which is significant for them [33], as such experiences of drug use or addiction could be a significant experience [34, 35]. IPA examines processes of personal meanings (instead of consequences), and how experience could change over time [33, 39]. Participants perceived SCs to be unpredictable and felt paranoid about the drug: their initial positive experiences quickly turned negative. They also reported that SCs took over their lives both interpersonally and intrapersonally: the drug took their olds friends away, and while initially it gave them new ones, in the end it not only made them asocial but the drug became their only friend. At last, it hijacked their personalities and made them addicted.

Unusual physical and psychological effects, psychotic and dependence symptoms, which were described by previous research [12, 16, 23], were also reported by the participants in this study. The appearance of negative effects happens rapidly; thus, participants barely recount positive experiences [6–8, 12, 16, 32]. The rapid development of tolerance, the experiences of addiction (e.g., craving and thoughts about smoking being the first things in the morning), lost control, and fears around adverse effects that we found in this study were also reported by Van Hout and Hearne [26].

According to participant accounts, the rapid development of negative experiences is the biggest difference between SCs and other drugs. In a qualitative study with people who used mephedrone [40], participants recalled mostly positive experiences (including euphoria, wellbeing, talkativeness). Adverse side effects were also reported as necessary components of the overall mephedrone experience, which was perceived as largely positive. Lee et al. [41] found that people who used ecstasy reported positive and predictable negative effects. The experience patterns of gamma hydroxybutyrate (GHB) reported by people who used the drug were also very similar [42]. The effects of GHB use were perceived mostly positive (such as euphoria, relaxation, increased sexual desire), but participants reported that negative effects were necessary in order to reach the desired effects of GHB. These risks could be controlled with the presence of a user group [42].

According to the accounts of participants in our study, the use of SCs evoked unpredictable and severe effects such as psychosis, as it was also described by Every-Palmer [29]. As such, the consumption of SCs could cause not only temporal psychotic symptoms but also persistent ones [30]. Due to the rapid alteration of experiences and psychotic symptoms, participants perceived the effects of SCs unpredictable, which explains the paranoid perceptions. It is important to note that we did not have information about what kind of SCs participants use during their drug consumption—usually neither the people who use nor the dealers know what actual compounds are on the market. This also could be a factor of unpredictability. Furthermore, the changing experience of positive to negative effects could be related to legislative changes that have led to more toxic SCs being used to make the products. As Barratt et al. [9] outlined, JWH-018 did not appear to have any more toxicity or likelihood to cause psychosis than natural cannabis. However, as Bright et al. [43] demonstrate, there is a complex interface between moral panic in the media, reactive legislation, and increased harm. This interplay between legislative changes and Hungarian media—where the portrayal of NPS could enhance moral panic [44, 45]—could contribute to the emergence of new SCs with increased toxicity.

Participants experienced a strange sense of self (the drug changed them, they became asocial, and the drug made them do things that they would have never done when they are sober) and they felt they were controlled or even hijacked by the drug. The narrative of a drug “taking over” one’s life is a personification of the drug (which is an old narrative of anti-drug propaganda, see: [46, 47]), may have been used here as a rationalization or justification of their own problematic behavior.

Participants described asocial behavior as another impact of SC use. Though addiction in general is associated with a retreat from social connections and an avoidance of the outside world [48], people who used other drugs than SCs including mephedrone, ecstasy, and GHB reported increased sociability, talkativeness, and loss of social (and other) inhibitions due to the effects of the drugs [40–42]. However, isolation and turning inward seem to be the consequences of SC use, and therefore SC use is a potential mediator of asocial behavior [29]. Psychoactive substance user groups can function as a risk management strategy and a place to share the problems derived from drug consumption [40, 42, 49]. Although the presence of the user group could help to control the unpredictable effects of SCs, people who use SCs, however, often leave the user group.

Boserman [50] analyzed diaries of people who used cannabis and utilized IPA to explore experiences of cannabis use. When we compare those results with the experience of SCs use in this study, some similarities and some differences emerge. The experience of cannabis use is mostly positive and serves as a ritual or social and recreational action. Cannabis is regarded vital in order to re-equilibrate the lost balance of life. Due to fast alteration of experiences derived from SC use, however, participants in this study reported a predominance of negative experiences. The ritualistic approach provides a closer and intimate relationship with cannabis (the participants fondle and respect the drug), while users’ relationship with SCs is rather paranoid.

Since the toxicity profiles of NPS may be also very different to those of traditional drugs and hard to identify its health risks, and it is difficult to estimate its consumption levels, which may not be detected by conventional drug screens [51], it may be important to involve personal reports of NPS use in harm reduction and clinical treatment which are rather provide services according to “classical” drug harms [52]. The described experiences by the participants of the current study outlined the subjective aspect of SC harms including clinically significant withdrawal, acute mental health, and overdose symptoms (e.g., [25]) that reinforce the urgent need of harm reduction and treatment services’ enhanced preparedness.

Our study has several limitations. Based on the methodology of IPA, a small homogenous sample was recruited, which may question the study’s generalizability. In addition, only male participants attended, so our results may not apply to women. For this study, a purposive sample was recruited and consisted only of SC users who were in treatment (presumably they experienced problems with SC use). In addition, people who use SCs recreationally or who are not in treatment may understand their experiences differently. An additional important limitation is that our study participants had assumed but were unable to confirm that they had been using SCs. Therefore, the experiences may vary depending on whether they had actually been using SC or maybe another substance, e.g., URB-579 (see Nakajima et al. [53]) or various other chemicals that have been found on synthetic cannabis products ([13, 54]).

The narratives of drug use experience (such as drug “taking over” or “hijacking” personality) reflect the subjective views of the respondents, and this may be in part a result of the treatment setting, as well. Further research to explore the experiences of people who use SCs and not in treatment for drug problems is suggested. During the interviews, the participants of this study solely focused on the effects of the drug use, so an additional limitation of the study is the absence of information how other factors, such as individual factors, and biopsychosocial, social, and cultural contexts might shape the effects and harms of SC use. An additional limitation could be the absence of reports of other drug experiences which were not as emphatic in the accounts as experiences of SC use.

The importance of other factors in the examination of drug use is increasingly being recognized on research on other drugs. Duff [55] used the word “assemblage” to describe drug use as an act that is a network with many persons and highlighted the context’s impact on drug use practice and experience. The framework of “risk environment” developed by Rhodes [56] describes drug harms as products of social situations and environments in which individuals participate. These suggest the shift of responsibility for drug harms and the focus of harm reduction from the individual alone to social and political institutions which have a role in harm production. In Hungary, there is a noticeable growth of new psychoactive substance use, while availability of harm reduction services is very limited [4, 57].

## Conclusions

Our study suggests that the comparison of SCs to cannabis may be misleading: many people who use SCs, smoke them as an available alternative for cannabis and/or other drugs, but the use of SCs is often associated with more negative experiences (that are different from other drug experiences). Due to the rapid development of effects, participants had difficulties interpreting or integrating their experiences. Since these experiences are mostly unknown and unpredictable, a forum where people who use the drug could share their experiences could have a harm-reducing role (e.g., [58]). The rapid alteration of effects and experiences may explain the severe psychopathological symptoms, which may be important information for harm reduction and treatment services, where treatment staff should be aware of unpredictable mood changes.

From a harm reduction point of view, SC is underrepresented in harm reduction literature. Therefore, it is important to emphasize the impossibility of knowing the quantity, purity, or even the number of different SC compounds in a particular SC product (e.g., [59]). Another important aspect could emerge: people who use SCs do not (or rarely) access harm reduction services (while intravenous substance users visit these services, for example, needle exchange program, more often [4, 57]). People who use SCs rather utilize emergency and toxicology treatments only when they experience very adverse effects. Therefore, nurses of health care services have the possibility to give messages of harm reduction, for example, that people who use drugs should do it in a user company (to control its effects), or people who use drugs should consume them in smaller quantities each time. Also, staff of emergency treatment and toxicology has the possibility to offer people who use drugs a treatment spot in rehabilitation settings.

Our study findings suggest that despite of the adverse effects, including a rapid turn of experiences to negative, rapid development of addiction and withdrawal symptoms of SCs, participants continued using the drug because this drug was mostly available and cheap. Therefore, a harm reduction approach would be to make available and legal certain drugs that have less adverse effects and could cause less serious dependence and withdrawal symptoms, with controlled production and distribution (similarly to cannabis legalization in the Netherlands).

## Abbreviations

Bio: “Bio-weed” street name of SC in Hungary
GHB: Gamma hydroxybutyrate
IPA: Interpretative phenomenological analysis
NPS: New psychoactive substances
SCs: Synthetic cannabinoids
